# Alpha-defensins inhibit ERK/STAT3 signaling during monocyte-macrophage differentiation and impede macrophage function

**DOI:** 10.1186/s12931-023-02605-0

**Published:** 2023-12-11

**Authors:** Jungnam Lee, Naweed Mohammad, Yuanqing Lu, Regina Oshins, Alek Aranyos, Mark Brantly

**Affiliations:** Division of Pulmonary, Critical Care and Sleep Medicine, University of Florida, Gainesville, FL USA

**Keywords:** Alpha-1-antitrysin (AAT), AAT deficiency (AATD), α-defensin, ERK1/2, STAT3, CD163, CD206

## Abstract

Alpha-1-antitrypsin deficiency (AATD) is a genetic disorder associated with a 5–tenfold decrease in lung levels of alpha-1-antitrypsin (AAT) and an increased risk for obstructive lung disease. α-defensins are cationic broad-spectrum cytotoxic and pro-inflammatory peptides found in the azurophilic granules of neutrophils. The concentration of α-defensins is less than 30 nM in the bronchoalveolar lavage fluid of healthy controls but is up to 6 μM in AATD individuals with significant lung function impairment. Alveolar macrophages are generally classified into pro-inflammatory (M1) or anti-inflammatory (M2) subsets that play distinct roles in the initiation and resolution of inflammation. Therefore, monocyte-macrophage differentiation should be tightly controlled to maintain lung integrity. In this study, we determined the effect of α-defensins on monocyte-macrophage differentiation and identified the molecular mechanism of this effect. The results of this study demonstrate that 2.5 μM of α-defensins inhibit the phosphorylation of ERK1/2 and STAT3 and suppress the expression of M2 macrophage markers, CD163 and CD206. In addition, a scratch assay shows that the high concentration of α-defensins inhibits cell movement by ~ 50%, and the phagocytosis assay using flow cytometry shows that α-defensins significantly reduce the bacterial phagocytosis rate of monocyte-derived macrophages (MDMs). To examine whether exogenous AAT is able to alleviate the inhibitory effect of α-defensins on macrophage function, we incubated MDMs with AAT prior to α-defensin treatment and demonstrate that AAT improves the migratory ability and phagocytic ability of MDMs compared with MDMs incubated only with α-defensins. Taken together, this study suggests that a high concentration of α-defensins inhibits the activation of ERK/STAT3 signaling, negatively regulates the expression of M2 macrophage markers, and impairs innate immune function of macrophages.

**Supplementary Information:**

The online version contains supplementary material available at 10.1186/s12931-023-02605-0.

## Introduction

Defensins are small cysteine-rich cationic peptides of approximately 30 amino acids. They are broad-spectrum antimicrobial peptides that form an essential element of innate immunity. Defensins are classified into two subfamilies, α- and β-defensins, based on their disulfide bond linkages [1–3]. α-defensins are mainly produced by neutrophils, whereas β-defensins are produced by epithelial cells. The expression of defensins could be induced upon infection or inflammation [4, 5]. α-defensins are one of the major products secreted by activated human neutrophils [6]. They comprise 30 to 50% of the total protein content of the azurophilic granules of neutrophils, and activated neutrophils release up to 10% of their defensin content extracellularly [7]. Defensins are known to protect the host through their antimicrobial activities, but they can exert both negative and positive immunomodulatory effects, depending on their concentrations. At low concentrations, defensins are beneficial to host cells by promoting the clearance of invading pathogens, while at high concentrations, they are cytotoxic and induce the expression of proinflammatory cytokines. At concentrations exceeding 10 μg/ml, α-defensins increase the expression levels of CXCL5, CXCL8 and IL-1β in lung epithelial cells. At a concentration of 20 μg/ml, α-defensins reduce the viability of lung epithelial cells by 30% [4, 8].

Alpha-1 antitrypsin (AAT) is the most abundant serine protease inhibitor in human plasma and plays an important role in limiting lung injury triggered by proteases such as neutrophil elastase [9, 10]. AAT deficiency (AATD) results from mutations in the SERine Protein INhibitor-A1 (*SERPINA1*) gene [11]. A hallmark of AATD is the accumulation of alveolar neutrophils, which leads to a high concentration of neutrophil products including α-defensins [12, 13]. As the number of alveolar neutrophils is significantly higher in AATD individuals, the concentration of α-defensins is highly increased in the lower respiratory tract. The concentration of α-defensins is less than 30 nM in the lower respiratory tract of healthy controls but is, on average, 2 μM in that of AATD individuals with mild lung disease [7]. The concentration is increased to 6 μM in the lower respiratory tract of AATD individuals with more severe lung function impairment [14]. This suggests that the concentration of α-defensins could be correlated to the severity of lung diseases in AATD individuals. However, it is still unclear what role α-defensins play in the pathogenesis of AATD-associated lung disease.

Alveolar macrophages reside at the interface between air and lung tissue, serving as the front line of cellular defense against respiratory pathogens. Macrophage differentiation occurs in concomitance with the acquisition of functional phenotypes, and the ability of macrophages to obtain different functional phenotypes allows them to respond with appropriate immune functions. During lung inflammation, airway macrophages are depleted and replaced by recruited monocytes that differentiate to macrophages [15]. Depletion of alveolar macrophage increases the severity of acute inflammation, pulmonary neutrophil infiltration, lung tissue damage, and sepsis [16, 17]. Thus, monocyte-macrophage differentiation is critical in regulating the inflammatory response and in curtailing inflammation during the resolution phase of lung inflammation. It was previously found that the number of alveolar macrophages is significantly decreased in the lower respiratory tract of AATD individuals, and α-defensins inhibit M-CSF–induced macrophage differentiation, contributing to the pathogenesis of chronic myelomonocytic leukemia [14, 18]. Based on those findings, we suspect that a high concentration of α-defensins could suppress monocyte-macrophage differentiation, which could lower the number of alveolar macrophages and impair innate immunity in AATD individuals.

Extracellular signal-regulated kinase 1/2 (ERK), a component of the mitogen-activated protein kinase (MAPK) family, controls cell proliferation and cell development by the transmission of extracellular signals to intracellular targets [19, 20]. ERK 1/2 signaling is activated by growth factors, and activated ERK signaling plays a critical role during macrophage differentiation and polarization [21]. STAT3 is a downstream molecule of ERK1/2 [22]. The activation of ERK1/2 and STAT3 signaling is important in M2 macrophage differentiation [23, 24]. STAT3 is a transcription factor that regulates the expression of M2 macrophage markers such as CD163 and CD206 [25, 26]. Several studies were previously conducted to examine the effect of α-defensins on the phosphorylation of ERK1/2 and reported that α-defensins induce the phosphorylation of ERK1/2. However, all the studies were carried out using cancer cell lines such as A549, U937, and HT-29 and treated the cells with α-defensins for a very short period, 30 min, or treated the cells with too high a concentration of α-defensins, 50 µg/ml [27, 28]. To determine the role of α-defensins at the concentration found in the lower respiratory tract, we examined the effect of α-defensins on the phosphorylation of ERK1/2 using experimental conditions which are biologically relevant for AATD individuals.

In the present study, we investigated the effect of α-defensins on monocyte-macrophage differentiation and therefore compared the expression levels of macrophage markers between controls and α-defensin-treated cells. We determined that α-defensins increase the expression level of CD80, an M1 macrophage marker, but reduce the expression levels of CD163 and CD206, M2 macrophage markers, during M-CSF and GM-CSF-derived macrophage differentiation. We also compared the levels of phosphorylated ERK1/2 and STAT3 between controls and α-defensin-treated cells and determined that the levels of phosphorylated ERK1/2 and STAT3 are significantly decreased as the concentration of α-defensins is increased. This observation might explain the inhibitory effect of α-defensins on M2 macrophage differentiation. Our results show that a high concentration of α-defensins significantly reduces the migratory ability and the phagocytic capability of MDMs. This indicates that a high concentration of α-defensins could negatively modulate innate immunity of macrophages in AATD individuals. AAT binds to α-defensins and neutralizes their cytotoxic effects on bronchial epithelial cells [29, 30]. In this study, we demonstrate that exogenous AAT at least partially alleviates the inhibitory effect of α-defensins on macrophage motility and phagocytosis. This result substantiates that AAT augmentation therapy might mitigate α-defensin-associated lung diseases. It is essential to understand the inhibitory effects of α-defensins on lung homeostasis and evaluate the efficacy of AAT against α-defensins to complement currently available therapies or develop more effective therapies for AATD-associated lung disease. Due to the general thought that α-defensins are beneficial to host cells, the role of α-defensins in the pathogenesis of AATD-associated lung disease has been seldom studied. The findings of this study bring new insights into the pathogenesis of α-defensin-associated lung disease in AATD individuals and serve to identify a potential therapeutic target to reduce the burden of lung disease in AATD individuals.

## Materials and methods

### Monocyte isolation and macrophage differentiation

Peripheral blood mononuclear cells (PBMCs) were isolated either from Leukopaks (obtained from LifeSouth Community Blood Center, Gainesville, FL) or blood samples of outpatient volunteers (University of Florida Institutional Review Board protocol 2015-01051), using Ficoll-gradient centrifugation. Monocytes were purified from PBMCs using a monocyte enrichment kit (Stemcell Technology, Vancouver) following the manufacturer’s instruction. Monocytes were plated in 12-well plates at one million cells per well and incubated in RPMI 1640 containing 10% FBS, 100 Units/ml penicillin, 100 μg/ml streptomycin, and 250 ng/ml Amphotericin B overnight. The stabilized monocytes were incubated in macrophage differentiation media (RPMI 1640 containing recombinant human GM-CSF (0.5 ng/ml) and recombinant human M-CSF (5 ng/ml)) in the presence or absence of α-defensins for 16 h. MDMs were harvested for RNA extraction using the Qiagen RNeasy kit (Qiagen, Hilden).

### Gene expression by qRT-PCR

Total RNAs (500 ng) extracted from monocytes and MDMs were reverse transcribed using SuperScript® VILO Master Mix (Invitrogen, Carlsbad) according to the manufacturer’s instruction. Quantification of PCR products was performed by 7500 Fast Real-time PCR (Applied Biosystems, Foster City). SensiFAST Real-Time PCR Kit (Bioline, London) was used to produce fluorescence-labeled PCR products and to monitor increasing fluorescence during repetitive cycling of the amplification reaction. TaqMan probes/primers specific for CD64, CD80, CD86, CD163, CD204, and CD206 genes, and for the 18S rRNA gene, as the internal control, were used in the real-time PCR reaction. Expression levels of the genes were obtained using the classical 2^(-ΔΔCt) method.

### Flow cytometry

The percentage of CD206-positive cells were compared among monocytes, MDM controls, and 2.5 μM of α-defensin-treated MDMs using flow cytometry. Cells were washed with PBS and centrifuged at 350 g for 5 min at room temperature. The cell pellet was resuspended in 100 μl of PBS and incubated with 5 μl of BV421 mouse anti-human CD206 at 4 °C for 30 min, protected from light. After the incubation, the labeled cells were fixed with 4% paraformaldehyde at 4 °C for 30 min. The fixed cells were washed with PBS and analyzed on a BD FACSCanto II instrument (BD Biosciences). 10,000 events were acquired per sample. The percentage of CD206-positive cells was measured by the BD FACSCanto II flow cytometer with BD FACSDiva 8.0.1 software (BD Biosciences).

### Western blot analysis

Total proteins were extracted from MDMs using RIPA lysis buffer (Cell Signaling Technology, Danvers) with 0.1% SDS, protease and phosphatase inhibitors. The protein concentration of each sample was measured using a standard Bradford assay (BioRad, Hercules) and equal amounts of protein samples were loaded onto an SDS polyacrylamide gel (BioRad, Hercules). After gel electrophoresis, the proteins were transferred onto a nitrocellulose membrane using a wet-transfer system, and the membrane was blocked in Tris-buffered saline with 0.1% Tween 20 (TBST) containing 5% nonfat dry milk. When detecting the phosphorylated form of any target proteins, Tris-buffered saline with 0.1% Tween 20 (TBST) containing 5% BSA was used as a blocking solution. The membrane was immunoblotted overnight at 4 °C with primary antibodies: CD163 (Novus Biologicals, Littleton), ERK1/2, phosphor-ERK1/2 and phosphor-STAT3 (Cell Signaling Technology, Danvers) at a dilution of 1:1,000 in TBST. Horseradish peroxidase-conjugated anti-rabbit antibody (BioRad, Hercules) was used for secondary labeling at 1:1,000 in TBST for 1 h at room temperature. The membrane was reprobed with GAPDH rabbit polyclonal antibody (Proteintech, Rosemont) at 1:5,000 in TBST. A horseradish peroxidase-conjugated anti-rabbit (BioRad, Hercules) was used for secondary labeling at 1:5,000 in TBST for 1 h at room temperature. Protein bands were visualized by enhanced chemiluminescence (ECL, GE Healthcare, Chicago).

### Trypan blue staining

Monocytes were plated in 12-well plates at 300,000 cells per well and differentiated in macrophage differentiation media. At day 7 of macrophage differentiation, MDMs were incubated in the presence or absence of α-defensins (Anaspec, Fremont) for 16 h. Trypan blue staining was used to measure cell membrane integrity. MDMs were incubated with 0.1% of trypan blue for three minutes and washed with PBS three times. The images of three different groups, MDM controls, 1 μM of α-defensin-treated MDMs, and 2.5 μM of α-defensin-treated MDMs, were taken using a light microscope at 40X magnification. The number of trypan-blue positive cells were counted and compared among the three different MDM groups.

### MTT assay

Cell viability of MDMs, which were incubated with or without α-defensins, was measured using a 3-(4,5-dimethylthiazol-2-yl)-2,5-diphenyltetrazolium bromide (MTT) assay. After α-defensin treatment, cells were washed with PBS to remove the extracellular particles and treated with MTT (0.5 mg/ml) for 30 min at 37 °C. Subsequently, the media containing the MTT reagent was removed and replaced with dimethyl sulfoxide (DMSO). The cell culture plate was incubated for five minutes to solubilize formazan crystals. Each well was pipetted again to mix, and absorbance at 570 nm was measured using a microplate reader (SpectraMax M3 ROM v3.0.22). Cell viabilities of α-defensin-treated MDMs were normalized to that of MDM controls.

### Scratch assay

Monocytes were plated in 12-well plates at 300,000 cells per well and differentiated in macrophage differentiation media. At day 7 of macrophage differentiation, the scratch was performed in the well with pipette tips and washed with PBS. Subsequently, the cells were cultured in RPMI 1640 media with or without α-defensins for 16 h. The number of cells which migrated into the scratched area was counted and compared between MDM controls and α-defensin-treated MDMs.

### Bacterial phagocytosis by MDMs

Heat-killed *Staphylococcus aureus* conjugated with Alexa Fluor 488 (ThermoFisher S-23371) was used to examine the phagocytic ability of MDMs. Lyophilized bacteria were dissolved in PBS with 2 mM sodium azide using sonication. MDMs were treated with α-defensins for 16 h. MDM controls and α-defensin-treated MDMs were incubated with the heat-killed fluorescent bacteria at a multiplicity of infection (MOI) of 10 for one hour. The non-ingested bacteria were removed by repeated washing with PBS. MDMs were incubated in 0.4% trypan blue solution for ten seconds to quench the fluorescence of bacteria attached to the plasma membrane of MDMs. Phagocytosed bacteria were visualized using a fluorescence microscope (BZ-X700, Keyence, Osaka). The cell membrane of live MDMs was stained using MemGlow™ 560 fluorogenic probes (Cytoskeleton, Denver). To calculate the phagocytosis rate of each MDM sample, fluorescent intensity and number of MDMs were measured with BZ software, and the fluorescent intensity was normalized to the cell number. To examine the effect of exogenous AAT on bacterial phagocytosis by α-defensins-treated MDMs, MDMs were incubated with 2.5 μM or 10 μM of AAT prior to α-defensin treatment. MDM samples were then incubated with the heat-killed fluorescent bacteria at an MOI of 10 for one hour. The phagocytosis rate of MDMs was analyzed by CytoFLEX flow cytometer with CytExpert software (Beckman Coulter, Brea). A minimum of 20,000 events were acquired per sample.

### AAT treatment

Lyophilized AAT (Prolastin-C) was reconstituted with deionized water, following the manufacturer’s instruction, and stored at -80 °C. To examine whether AAT is able to alleviate the inhibitory effect of α-defensins on cell motility and the phagocytic ability of MDMs, MDMs were incubated with 2.5 μM or 10 μM of AAT prior to α-defensin treatment.

### Statistical analysis

Results are expressed as mean and standard deviation or percentage as appropriate. Comparisons between groups were made by using non-parametric tests, Wilcoxon matched-pairs signed rank test or one-way analysis of variance (ANOVA). A p-value < 0.05 was considered significant. All analyses were performed using the GraphPad Prism 9.3.0 (GraphPad software, San Diego) software package.

## Results

### The effect of α-defensins on monocyte-macrophage differentiation

**Fig. 1:**
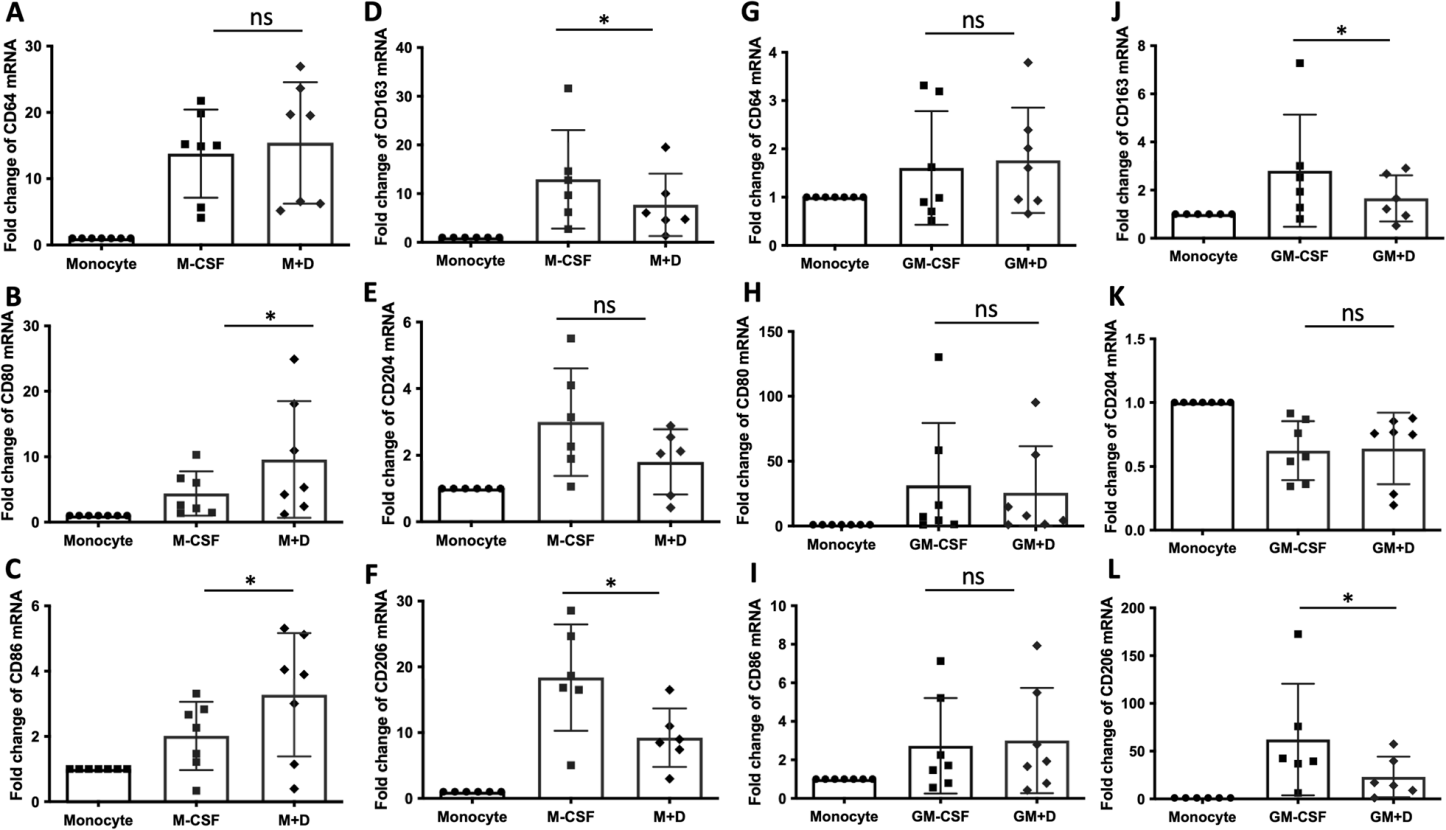
The effect of α-defensins on macrophage differentiation. MDMs were differentiated using either M-CSF or GM-CSF and incubated with 2.5 µM of α-defensins for 16 h. **A**–**C** The expression levels of M1 macrophage markers, CD64, CD80, and CD86, were compared among three different groups: monocyte, M-CSF-treated monocyte (M-CSF), and M-CSF and α-defensin-treated monocyte (M + D). Their relative expression is represented by fold change. **D**–**F** The expression levels of M2 macrophage markers, CD163, CD204, and CD206, were also compared among the groups (monocyte, M-CSF, and M + D). **G**–**I** The expression levels of M1 macrophage markers were compared among three different groups: monocyte, GM-CSF-treated monocyte (GM-CSF), and GM-CSF and α-defensin-treated monocyte (GM + D). Their relative expression is represented by fold change. **J**–**L** The expression levels of M2 macrophage markers were also compared among the groups (monocyte, GM-CSF, and GM + D). Statistical analysis was conducted using Wilcoxon test. Statistical significance is denoted by (*) (p-value < 0.05)

M-CSF and GM-CSF are hematopoietic growth factors. M-CSF is ubiquitously produced by many cells and controls macrophage numbers in many tissues. M-CSF-derived macrophages are often used as a model for tissue macrophages. GM-CSF has a low basal circulating level but is elevated during inflammatory reactions. GM-CSF is essential for alveolar macrophage differentiation and for maintenance of alveolar macrophage functions throughout life [31, 32]. Because M-CSF and GM-CSF have different functions, we wanted to examine the effect of α-defensins on M-CSF-derived macrophage differentiation and GM-CSF-derived macrophage differentiation separately. To examine the effect of α-defensins on the initiation of monocyte-macrophage differentiation, we incubated monocytes with M-CSF for 16 h and examined the expression levels of M1 macrophage markers CD64, CD80, and CD86, and M2 macrophage markers CD163, CD204, and CD206 [33]. The expression levels of the macrophage markers were highly increased by M-CSF, as shown in Fig. 1A–F. When cells were incubated with M-CSF in the presence of 2.5 μM of α-defensins, the expression levels of M1 macrophage markers CD80 and CD86 were significantly increased by α-defensins (p-value = 0.0312 and p-value = 0.0469, Fig. 1B, C, respectively). The expression levels of M2 macrophage markers, CD163 and CD206, were significantly reduced by α-defensins during M-CSF-derived macrophage differentiation (p-value = 0.0312 and p-value = 0.0312, Fig. 1D, F respectively). The expression level of CD204 was also reduced by α-defensins, but the decrease was not significant (Fig. 1E). To examine the effect of α-defensins on GM-CSF-derived macrophage differentiation, monocytes were differentiated in the presence of GM-CSF, and the expression levels of the three different M1 macrophage markers and the three different M2 macrophage markers were compared among monocytes, GM-CSF-treated cells, and GM-CSF and α-defensin-treated cells. GM-CSF also increased the expression levels of all the M1 macrophage markers, CD64, CD80, and CD86, during the monocyte-macrophage differentiation, but α-defensins had no effect on the expression of M1 macrophage markers during GM-CSF-derived macrophage differentiation (Fig. 1G–I). GM-CSF induced the expression levels of M2 macrophage markers, CD163 and CD206, but α-defensins significantly reduced the expression levels of CD163 and CD206 during GM-CSF-derived macrophage differentiation (p-value = 0.0312 and p-value = 0.0312, Fig. 1J and L). Therefore, regardless of the growth factor, α-defensins consistently suppressed the expression of M2 macrophage markers CD163 and CD206 during monocyte-macrophage differentiation.

**Fig. 2:**
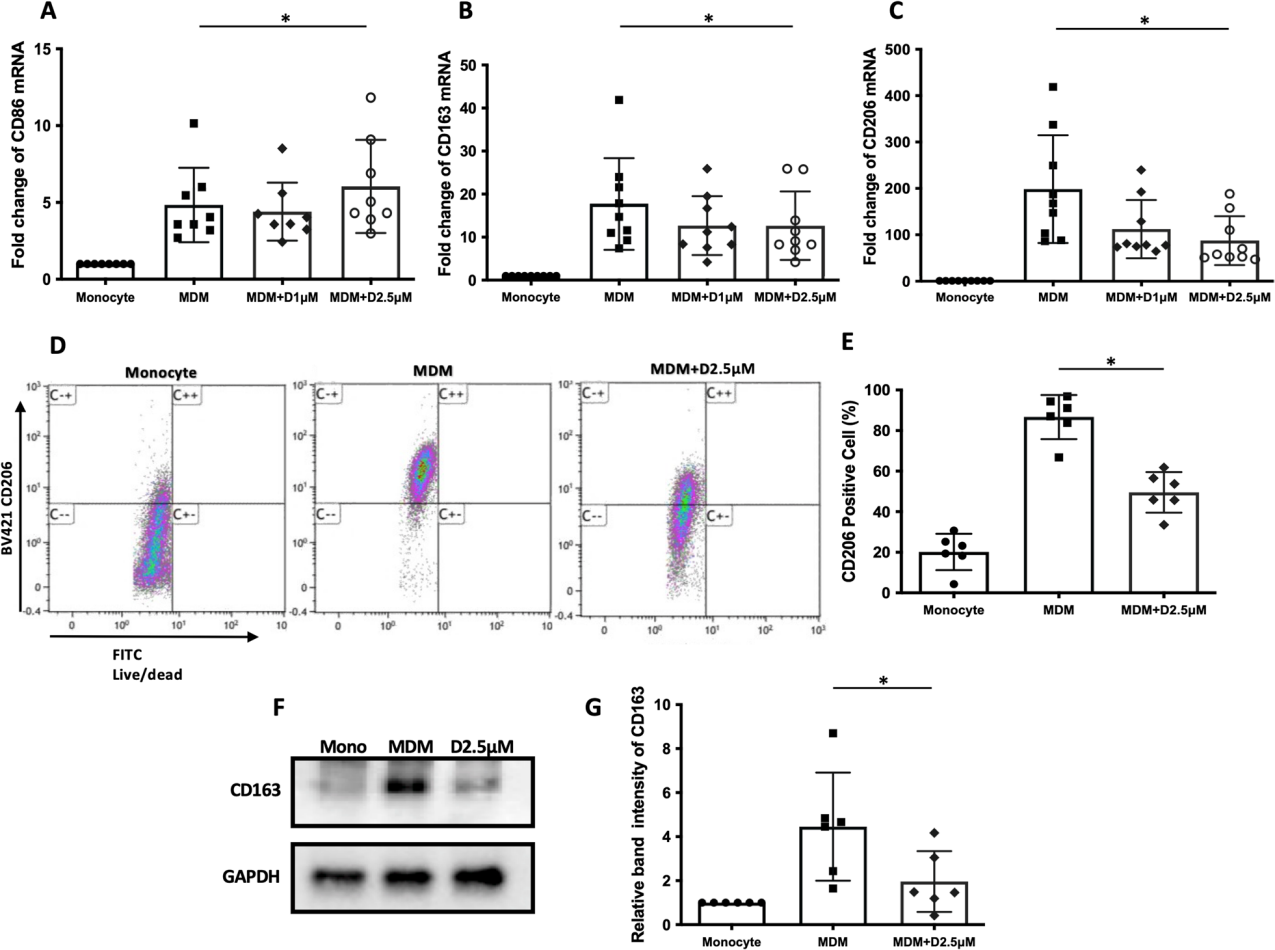
The effect of α-defensins on M-CSF and GM-CSF-induced macrophages. MDMs were differentiated using both M-CSF and GM-CSF and incubated with two different concentrations of α-defensins, 1 µM and 2.5 µM, for 16 h. **A** The expression level of CD86 was compared between MDM controls and α-defensin-treated MDMs. **B**, **C** The expression levels of CD163 and CD206 were compared between the two MDM groups. The relative expression of the macrophage markers is represented by fold change. Statistical analysis was conducted using One-way ANOVA. Statistical significance is denoted by (*) (p-value < 0.05). The protein levels of the M2 macrophage markers were also examined in α-defensin-treated MDMs. **D** Cell surface distribution of CD206 was examined using flow cytometry. **E** The percentage of CD206-positive cells was compared between MDM controls and α-defensin-treated MDMs. **F** The protein level of CD163 was examined using a Western blot assay. **G** The protein band intensities were measured using NIH ImageJ software and compared between MDM controls and α-defensin-treated MDMs. Statistical analysis was conducted using Wilcoxon test. Statistical significance is denoted by (*) (p-value < 0.05)

Because monocytes are differentiated into alveolar macrophages in the presence of both M- and GM-CSF in the pulmonary alveoli, it was intriguing to examine the effect of α-defensins on monocyte-macrophage differentiation when monocytes were incubated with M- and GM-CSF together. The result shows that α-defensins significantly increased the expression level of CD86 (p-value = 0.0464, Fig. 2A), but the expression levels of M2 macrophage markers CD163 and CD206, were significantly decreased as the concentration of α-defensins were increased during M- and GM-CSF-derived monocyte-macrophage differentiation (p-value = 0.0075 and p-value = 0.008, Fig. 2B, C, respectively). Taken together, the results propose that a high concentration of α-defensins could modulate monocyte-macrophage differentiation by affecting the expression of macrophage markers in the lower respiratory tract of AATD individuals. Figures 1 and 2 focus on the effect of α-defensins on the initiation of the process of monocyte-macrophage differentiation. It was intriguing to examine the effect of α-defensins in MDMs, which were differentiated with M- and GM-CSF for seven days. We found that α-defensins significantly reduce the expression of CD206 in MDMs (Additional file 1: Fig. S1). This suggests that α-defensins could modulate monocyte-macrophage differentiation not only in the initiation of the process, but also that it could modify the phenotypes of differentiated macrophages. On the other hand, we compared the inhibitory effect of α-defensins on the expression of CD206 between MDMs with the M-AAT allele (M-MDM) and with the Z-AAT allele (Z-MDM). The Z variant is responsible for the most severe form of AAT deficiency. This result showed that α-defensins reduce the gene expression level of CD206 in both M- and Z-MDMs, and the reduced expression level was similar between the two MDM groups (Table 1). The sample number was six for each group, and none of the individuals had lung disease when blood was collected. The characteristics of the control and AATD individuals used in Table 1 were previously described [34].

**Table 1:** The expression of CD206 reduced by α-defensins

	D1μM (%)	*P*-value	D2.5 μM (%)	*P*-value
M-MDM	34.9 ± 26	0.73	53 ± 17	0.891
Z-MDM	41.6 ± 6.6		51 ± 9.8	

### The protein levels of CD206 and CD163 reduced by α-defensins

Alveolar macrophages are characterized by a high level of CD206 and CD163 [35]. CD206 is a mannose receptor primarily expressed on the surface of macrophages. CD206 on the surface of alveolar macrophages interacts with glycoproteins and glycolipids found on the surface of invading pathogens, including bacteria, viruses, and fungi. Therefore, CD206 plays an important role in the innate immunology of macrophages. We compared the cell surface levels of CD206 between MDM controls and α-defensin-treated MDMs using flow cytometry. The percentage of CD206-positive cells was highly increased by growth factors M- and GM-CSF, but significantly reduced when the cells were incubated with 2.5 μM of α-defensins, as shown in Fig. 2D, E (p-value = 0.0312). CD163 is a scavenger receptor and has a capacity for ligand binding and endocytosis [35]. Thus, CD163 functions as an innate immune sensor for gram-positive and gram-negative bacteria [36]. We tried to examine the effect of α-defensins on the cell surface distribution of CD163 using flow cytometry, but flow cytometry did not work for this purpose because the human genome contains the CD163-L1 gene, which was produced by duplication of CD163 in late evolution [37]. Therefore, CD163 antibody binds to both CD163 and CD163-L1. Instead of flow cytometry, we performed Western blotting to examine the effect of α-defensins on the protein level of CD163. Because the molecular weights of the two proteins are different, CD163 was separated from CD163-L1 on the SDS gel. The result shows that, consistently with the gene expression of CD163, the protein level of CD163 was highly increased by growth factors but significantly reduced by 2.5 μM of α-defensins, as shown in Fig. 2F, G (p-value = 0.0312).

### The activation of ERK/STAT3 signaling inhibited in α-defensins-treated cells

**Fig. 3:**
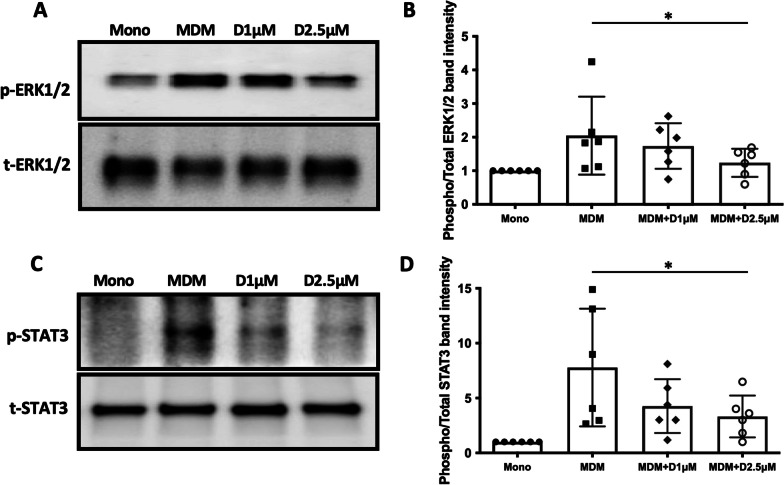
The phosphorylation of ERK1/2 and STAT3 inhibited by α-defensins. Total proteins were isolated from monocytes, MDM controls, and α-defensin-treated MDMs. **A** Equal amounts of total proteins of the four different samples were analyzed via SDS-PAGE of phosphorylated ERK1/2 and total ERK1/2. **C** Equal amounts of total proteins of the four different samples were analyzed via SDS-PAGE of phosphorylated STAT3 and total STAT3. **B** and **D** The protein band intensities of the phosphorylated ERK1/2 and phosphorylated STAT3 were measured using NIH ImageJ software and compared among the samples. Statistical analysis was conducted using Wilcoxon test. Statistical significance is denoted by (*) (p-value < 0.05)

It was previously suggested that BmKDfsin3, one of the scorpion defensins, inhibits the phosphorylation of p38 [38]. p38 is well known for playing a key role in macrophage polarization [39]. BmKDfsin3 has six cysteine residues and the cysteine residues form three intramolecular disulfide bridges, which is the same as α-defensins. Therefore, we suspected that α-defensins suppress the expression of M2 macrophage markers by inhibiting the phosphorylation of p38 during M- and GM-CSF-derived macrophage differentiation. To address this question, we differentiated MDMs using M- and GM-CSF in the absence or presence of α-defensins and compared the level of phosphorylated p38 between MDM controls and α-defensin-treated MDMs. The level of phosphorylated p38 was highly increased by the growth factors. However, unlike the scorpion defensin, α-defensins did not decrease the level of phosphorylated p38 during monocyte-macrophage differentiation (Additional file 1: Fig. S2). Besides p38 signaling, ERK1/2 is an essential molecule for growth factor-driven macrophage proliferation, survival, or differentiation [21]. It was previously found that the activation of ERK signaling enhances macrophage phenotypic polarization from proinflammatory M2b to anti-inflammatory M2a [40]. To examine whether ERK1/2 is involved in α-defensin-suppressing M2 macrophage marker expression, we compared the level of total ERK1/2 and the level of phosphorylated ERK1/2 among monocytes, MDM controls, MDMs incubated with 1 μM of α-defensins, and MDMs incubated with 2.5 μM of α-defensins. The level of total ERK1/2 was very similar across the four different samples as shown in Fig. 3A. This indicates that α-defensins have no effect on the transcription and translation of ERK1/2. Figure 3A also shows the level of phosphorylated ERK1/2 in the four different samples. The level of phosphorylated ERK1/2 was highly increased by the growth factors but significantly decreased by α-defensins during M- and GM-CSF-derived macrophage differentiation (p-value = 0.0312, Fig. 3B). It is known that ERK1/2 is an upstream molecule of STAT3, which regulates the expression of CD163 and CD206. Indeed, the phosphorylation of ERK1/2 and STAT3 are important for M2 macrophage polarization. To examine whether α-defensins inhibit the phosphorylation of STAT3, we compared the levels of phosphorylated STAT3 among the four different samples. The result shows that the level of phosphorylated STAT3 was highly increased by M- and GM-CSF but significantly decreased by α-defensins (p-value = 0.0312, Fig. 3C, D). Figure 3B shows that the protein level of total STAT3 was not reduced by α-defensins. Therefore, the result suggests that α-defensins inhibit the phosphorylation of STAT3 in MDMs. U0126 inhibits the activation of ERK1/2 [41]. We incubated MDMs with U0126 and confirmed that the phosphorylation of STAT3 is inhibited when the phosphorylation of ERK1/2 is inhibited by U0126. The protein level of CD163 and the expression level of CD206 were also decreased in U0126-treated MDMs (Additional file 1: Fig. S3). This indicates that ERK1/2 signaling regulates the phosphorylation of STAT3 and the expression of CD163 and CD206 in M- and GM-CSF-derived macrophages.

### Membrane damage using trypan blue staining

**Fig. 4:**
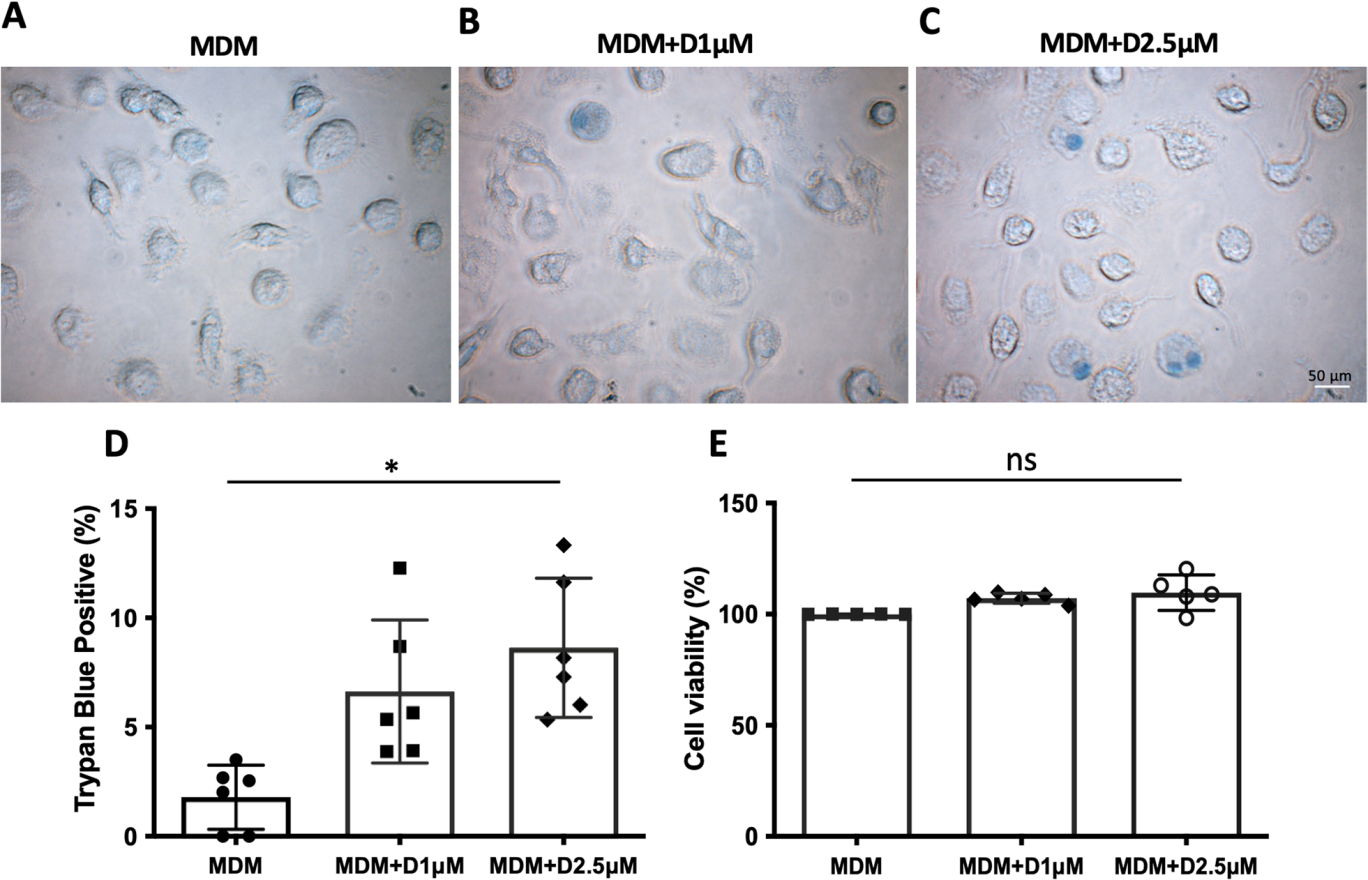
Cell viability of α-defensin-treated MDMs. MDMs were incubated with 1 µM or 2.5 µM of α-defensins for 16 h. **A**–**C** MDM controls and α-defensin-treated MDMs were stained with 0.1% of trypan blue for three minutes, and the images of trypan blue-stained MDMs were captured using a light microscope. **D** The percentage of trypan blue-positive cells was calculated and compared between samples. Statistical analysis was conducted using One-way ANOVA. Statistical significance is denoted by (*) (p-value < 0.05). **E** Cell viability of each MDM sample was measured using an MTT assay. Statistical analysis was conducted using One-way ANOVA, and (ns) indicates no statistical difference among the samples

Most defensins accomplish their antimicrobial activity through interaction with membranes of pathogens. Positively charged defensin peptides interact with the negatively charged head of phospholipid groups in cellular membranes when a critical defensin concentration is reached, which permeabilizes cell membranes of susceptible microorganisms. It was intriguing to examine whether a high concentration of α-defensins is able to permeabilize the cell membrane of MDMs. We assessed the membrane integrity of MDMs using trypan blue staining. Trypan blue is cell membrane impermeable. Therefore, it only enters cells when the plasma membrane is compromised. Trypan blue-positive cells were detected in α-defensin-treated MDMs, and the number of trypan blue-positive cells was significantly increased as the concentration of α-defensins was increased (p-value = 0.0026, Fig. 4A–D). It was previously reported that a high concentration of α-defensins is cytotoxic in lung epithelial cells [8]. To examine whether a high concentration of α-defensins is cytotoxic in MDMs, we measured and compared cell viability of MDM controls and α-defensin-treated MDMs using an MTT assay. The result shows that cell viability is very similar between MDM controls and α-defensin-treated MDMs (Fig. 4E). This indicates that even the highest concentration of α-defensins, 2.5 μM, did not induce cell death when MDMs were incubated with it for 16 h. This result is consistent with previous findings [42]. The authors of the study incubated lung epithelial cells with α-defensins (5 μg/ml) for 72 h and found that cell viability was not reduced by α-defensins, but about 15% of the cells were trypan blue-positive [43]. However, we could not rule out that α-defensins could cause cell death when MDMs are incubated either with concentrations of α-defensins higher than 2.5 μM or incubated with α-defensins for longer than 16 h.

### MDM migration after incubation with α-defensins

**Fig. 5:**
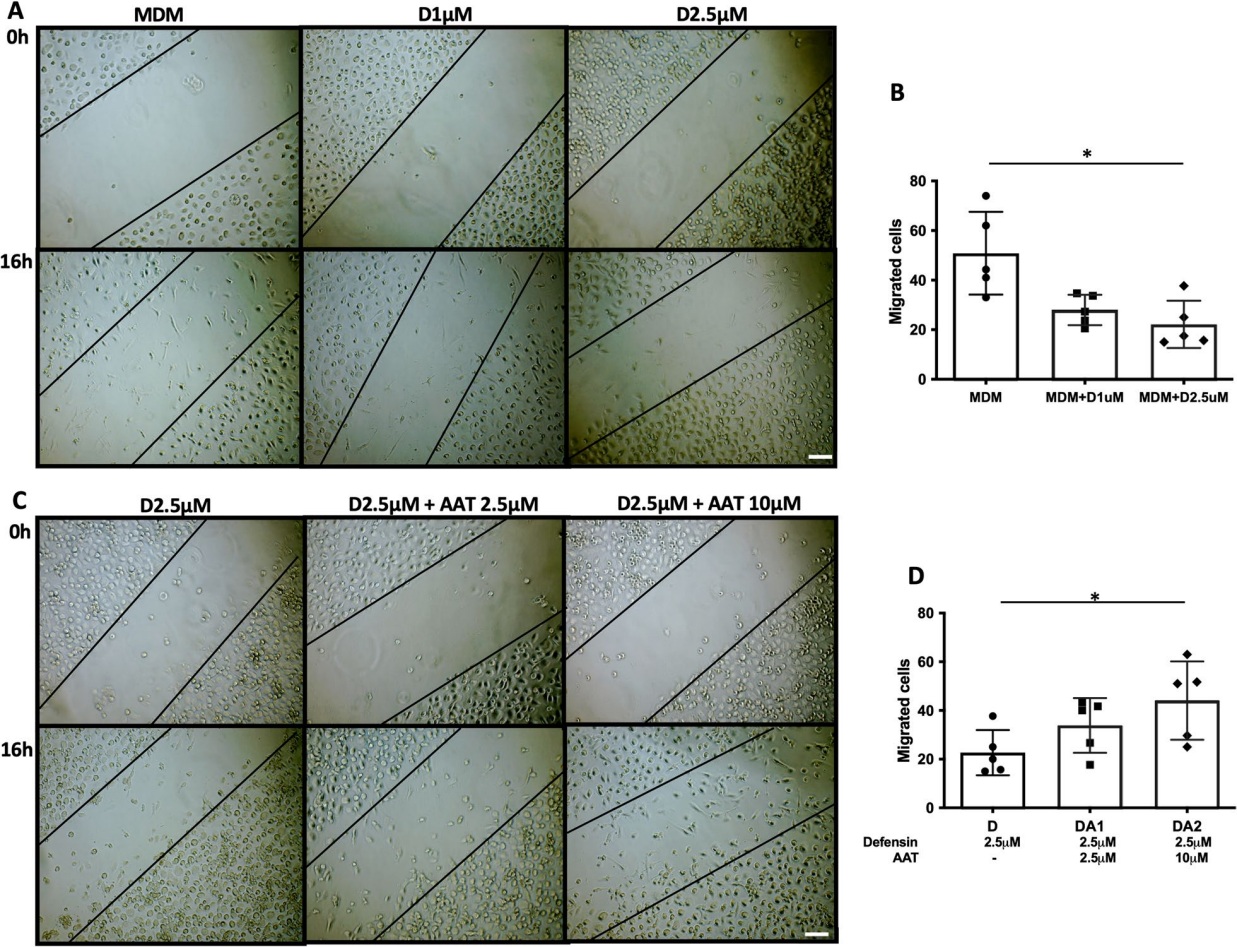
The effect of α-defensins on the migratory ability of MDMs. At day 7 of MDM differentiation, the scratch was performed. MDMs were incubated with or without α-defensins for 16 h. **A** The images of MDM controls and α-defensin-treated MDMs were taken using a light microscope; bar 200 µm. **B** The cells that moved into the scratched area were counted and compared among the three different MDM groups. **C** MDMs were incubated with 2.5 µM or 10 µM of AAT. After the incubation with AAT for one hour, MDMs were treated with 2.5 µM of α-defensins for 16 h. The images of the three different MDM groups (2.5 µM of α-defensin-treated MDMs, 2.5 µM of α-defensin and 2.5 µM of AAT-treated MDMs, and 2.5 µM of α-defensin and 10 µM of AAT-treated MDMs) were taken using a light microscope; bar 200 µm. **D** The cells that moved into the scratched area were counted and compared among the three different MDM groups. Statistical analysis was conducted using One-way ANOVA. Statistical significance is denoted by (*) (p-value < 0.05)

When MDMs were incubated with 1 μM or 2.5 μM of α-defensins, trypan blue was able to penetrate some of the α-defensin-treated MDMs. This indicates that a high concentration of α-defensins could cause cell membrane damage in MDMs. To examine whether α-defensins have an inhibitory effect on the migratory ability of MDMs, we conducted a scratch assay. After making a scratch, MDMs were incubated in the absence or presence of α-defensins for 16 h. The number of cells that migrated into the scratched area were counted and compared between MDM controls and α-defensin-treated MDMs. The result shows that the cell migration rate of MDMs was significantly decreased as the concentration of α-defensins was increased. 2.5 μM of α-defensins reduced the migratory ability of MDMs by 50% (p-value = 0.0015, Fig. 5A, B). This indicates that a high concentration of α-defensins suppresses the migratory ability of MDMs.

### Bacterial clearance by macrophages impaired by α-defensins

**Fig. 6:**
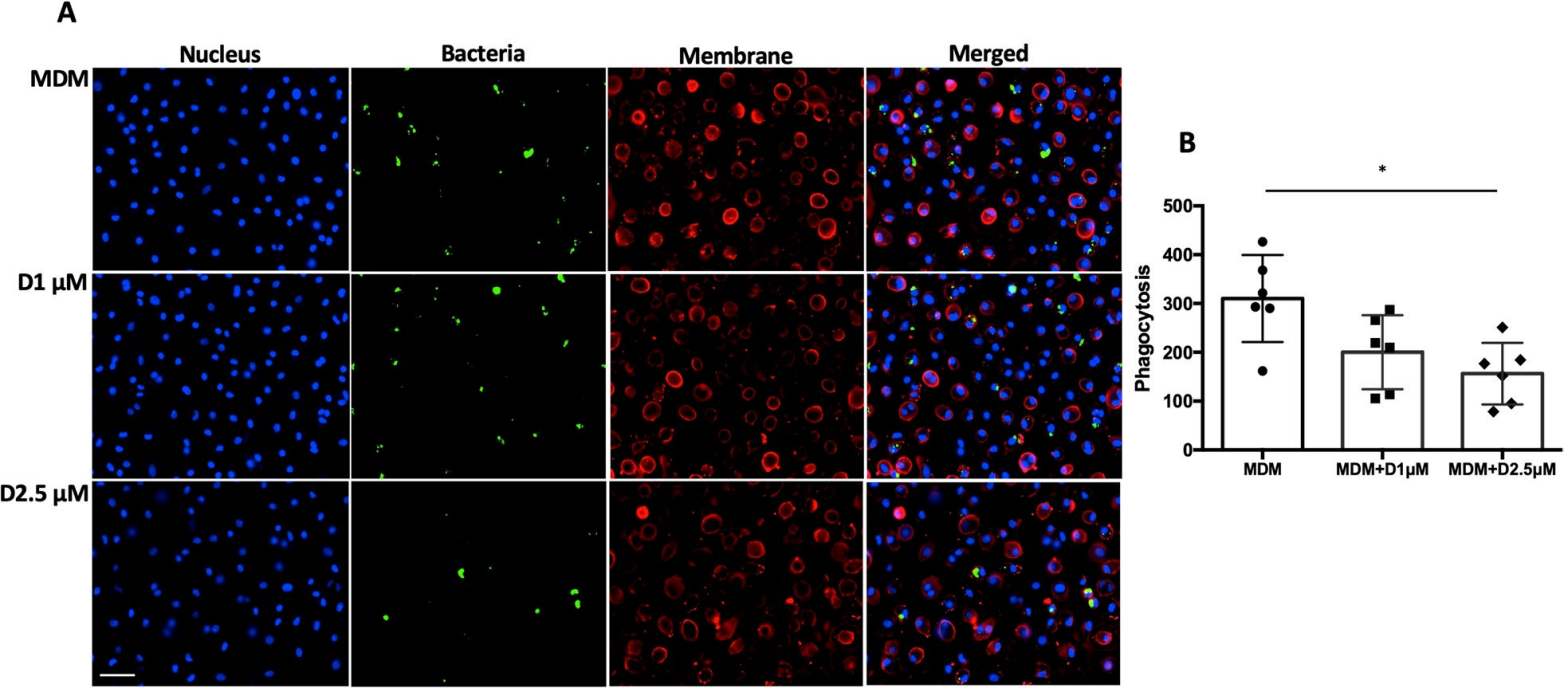
The bacterial phagocytosis of α-defensin-treated MDMs. MDMs were treated with 1 µM or 2.5 µM of α-defensins for 16 h and incubated with Heat-killed *Staphylococcus aureus*-conjugated with Alexa Fluor 488 at a multiplicity of infection (MOI) of 10 for one hour. **A** MDMs phagocytosing bacteria are visualized using a fluorescence microscope; bar 50 μm. Red and green indicate the host cell membrane and digested bacteria, respectively. **B** Green fluorescent intensity was normalized to the number of MDMs and compared among the different MDM groups. More than 10,000 cells, originating from six separate experiments, were evaluated for each MDM group. Statistical analysis was conducted using One-way ANOVA. Statistical significance is denoted by (*) (p-value < 0.05)

Alveolar macrophages are the primary phagocytes of the innate immune system, clearing bacteria from the lower respiratory tract. Therefore, their ability to clear invading pathogens is critical in maintaining lung homeostasis. The results of the present study show that cell migratory ability and the expression levels of CD163 and CD206 are reduced by α-defensins in MDMs. Cell motility is an essential feature for macrophages to phagocytose invading pathogens. CD163 and CD206 are also important for bacterial phagocytosis by macrophages. It was intriguing to examine the effect of α-defensins on bacterial phagocytosis by MDMs. MDMs were incubated in the absence or presence of α-defensins, and then the cells were incubated with fluorescently labeled heat-killed bacteria. The phagocytic ability of the MDMs was evaluated using fluorescent microscopy. The result shows that α-defensins suppress bacterial phagocytosis by MDMs, shown in Fig. 6A, B. One-way ANOVA found that the phagocytosis rate was reduced as the concentration of α-defensins was increased (p-value < 0.0001).

### Exogenous AAT helps recover α-defensin-causing macrophage impairment

**Fig. 7:**
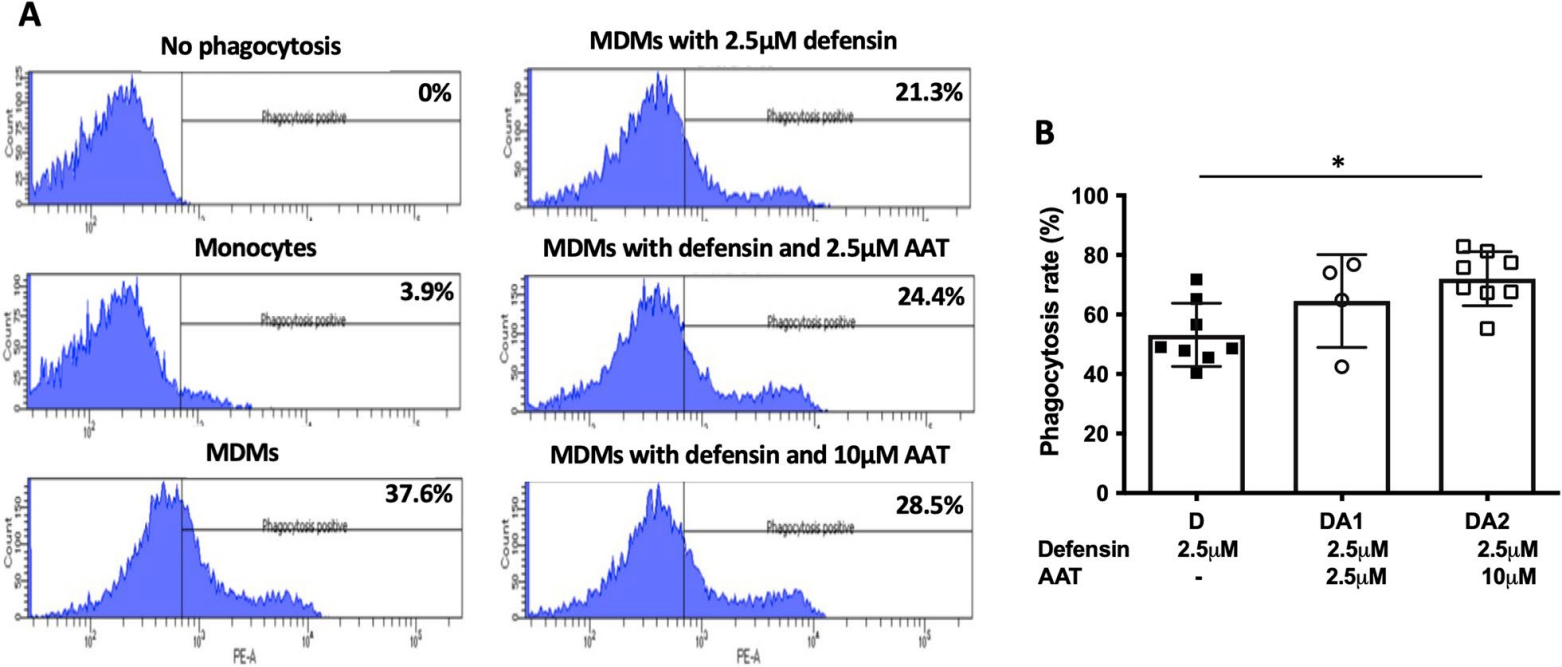
The phagocytic ability of α-defensin-treated MDMs improved by the AAT treatment. To examine the effect of exogenous AAT on α-defensin-induced injury to the MDM phagocytosis, six different samples were prepared, and without control (No phagocytosis), five samples were incubated with Heat-killed *Staphylococcus aureus-*conjugated with Alexa Fluor 488 at a multiplicity of infection (MOI) of 10 for one hour. The five different samples were monocytes, MDM controls, 2.5 µM of α-defensin-treated MDMs, 2.5 µM of α-defensin and 2.5 µM of AAT-treated MDMs, and 2.5 µM of α-defensin and 10 µM of AAT-treated MDMs. **A** The percentage of phagocytosis-positive cells was measured using flow cytometry. **B** To examine the effect of α-defensins on MDM phagocytosis, phagocytosis rates were compared among the four different MDM groups. The phagocytosis rate of MDM controls was set to 100%, and phagocytosis rates of the other MDM groups were normalized to the phagocytosis rate of MDM controls. Statistical analysis was conducted using One-way ANOVA. Statistical significance is denoted by (*) (p-value < 0.05)

Intravenous AAT suppresses neutrophil-mediated injury, and thus AAT augmentation therapy has been used to treat patients with AATD-associated diseases. It is known that AAT binds to α-defensins and neutralizes harmful effects of α-defensins on bronchial epithelial cells [29, 44]. This implies that exogenous AAT could alleviate the inhibitory effect of α-defensins on the migratory ability of MDMs. To examine that, we incubated MDMs either with α-defensins only or α-defensins and AAT together after making a scratch. After 16 h of incubation with the treatment, the number of cells that migrated into the scratched area was counted and compared between α-defensin-treated MDMs with and without AAT. Compared with MDMs incubated with 2.5 μM of α-defensins only, the migratory ability of MDMs improved with the addition of AAT, as shown in Fig. 5C. The migratory ability of MDMs was significantly increased as the concentration of AAT was increased (p-value = 0.0019, Fig. 5D). As exogenous AAT alleviated the inhibitory effect of α-defensins on the migratory ability of MDMs, it was worthwhile to examine whether exogenous AAT blocks the inhibitory effect of α-defensins on the phagocytic ability of MDMs. We incubated MDMs either with α-defensins alone or α-defensins and AAT together. Then, we incubated cells with heat-killed fluorescent bacteria and measured the phagocytosis rate of each sample using flow cytometry. 2.5 μM of α-defensins reduced the phagocytic ability of MDMs by 50%. However, 1 μM and 2.5 μM of AAT increased the phagocytosis rate of MDMs to 65% and 75%, respectively, as shown in Fig. 7A. One-way ANOVA found that the phagocytosis rate of MDMs was significantly increased as the concentration of AATs was increased (p-value = 0.0117, Fig. 7B).

## Discussion

The concentration of α-defensins is substantially higher in the lower respiratory tract of AATD individuals than control individuals, due to the accumulation of alveolar neutrophils, and the α-defensin concentration is increased as lung function impairs in AATD individuals [14]. Because α-defensins are well known for their antimicrobial property, it has been assumed that α-defensins are only beneficial to host cells. However, their antimicrobial activity is dependent on their environment. For example, α-defensins were very efficient to kill gram-positive and gram-negative bacteria in 10 mM phosphate buffer containing certain nutrients, but they had remarkably reduced bactericidal activity in nutrient-free buffer [1]. The antimicrobial activity of α-defensins in the lung has not been determined. Therefore, it is not clear whether the excessive amount of α-defensins helps to suppress bacterial infection in those lung diseases by killing the invading pathogens.

One of previous studies on α-defensins found that α-defensins could inhibit M-CSF-derived monocyte-macrophage differentiation. The study demonstrated that α-defensins inhibit the gene expression of CD163 and MCP1 in a dose-dependent manner during the M-CSF-derived macrophage differentiation. This suggests that α-defensins could modulate macrophage differentiation in the lung of AATD individuals. Because both growth factors, M- and GM-CSF, are present in the lower respiratory tract and GM-CSF is important for alveolar macrophage differentiation [32, 45], we examined the effect of α-defensins on M- and GM-CSF-derived microphage differentiation, and found that the expression level of M1 macrophage marker CD86 is significantly increased by α-defensins while the expression level of M2 macrophage markers CD163 and CD206 is significantly decreased by α-defensins. This might suggest that α-defensins could enhance M1 macrophage polarization but inhibit M2 macrophage polarization. The level of CD86 is significantly increased in patients with an acute asthma exacerbation and correlated with the severity of asthma [46]. In addition, the expression level of CD86 is increased after lung transplantation, and the number of CD86-positive alveolar macrophages is increased during rejection episodes [47]. Interestingly, it was previously reported that chronic stimulation of airway epithelial cells by α-defensins causes inflammation and fibrosis, leading to chronic rejection following human lung transplantation [48]. Therefore, the increased expression level of CD86 by α-defensins in macrophages could exacerbate airway diseases and promote chronic rejection of the new lung in AATD individuals. This study shows that the protein levels of both CD163 and CD206 are significantly reduced by α-defensins. It was experimentally proven that CD163 and CD206 are involved in phagocytosis of numerous strains of bacteria [49]. CD163 reduces iron bioavailability by mediating haptoglobin-hemoglobin uptake and hence could restrict bacterial growth in the pulmonary alveolus [50]. CD206 is a transmembrane pattern recognition receptor and plays a role in the phagocytosis of immune cells and bacteria as well as in pinocytosis through binding a variety of carbohydrates [26]. The result of this study suggests that a high concentration of α-defensins could add more risk for bacterial infection-mediated lung diseases by reducing the expression of CD163 and CD206 in AATD individuals. Interestingly, it was previously reported that AATD individuals are susceptible to recurrent bacterial infection which affects their respiratory tract [51].

There are accumulating data to support that α-defensins function as immunomodulatory molecules through P2Y6 signaling. One previous study on α-defensins found that α-defensins inhibit neutrophil apoptosis by acting on the P2Y6 receptor, and another study suggested that α-defensins induce the expression of IL-8 in lung epithelial cells through the P2Y6 signaling [52, 53]. Interestingly, it was suggested that the P2Y6 receptor plays an important role in M-CSF-derived macrophage differentiation [54], and α-defensins inhibit M-CSF-derived differentiation through P2Y6 [18]. The present study demonstrated that α-defensins reduce the gene expression and protein level of CD163 and CD206 by inhibiting the phosphorylation of ERK1/2. Given that ERK1/2 is a downstream molecule of the P2Y6 receptor [55], α-defensins might inhibit the phosphorylation of ERK1/2 through the P2Y6 receptor, suppressing M2 macrophage phenotypes during M- and GM-CSF-derived macrophage differentiation.

This study demonstrated that a high concentration of α-defensins causes cell membrane damage and consequently reduces the migratory ability of MDMs. Cationic antimicrobial peptides including α-defensins, target anionic lipids, including cardiolipin and phosphatidyl glycerol, on the cell surface, which are abundant in microorganisms. However, in the mammalian cell membrane, electrically neutral phospholipids, including sphingomyelin and phosphatidylcholine, are predominant. Therefore, the binding affinity of cationic antimicrobial peptides is much higher to the membrane of microorganisms than that of mammalian cells [56–58]. It might explain why a low concentration of α-defensins are beneficial to the host cells by killing invading pathogens, but a high concentration of α-defensins cause damage to the membrane of host cells. The present study found that a high concentration of α-defensins significantly reduced the phagocytic capability of macrophages. The migratory and phagocytic abilities of macrophages are critical for lung homeostasis. The lung is constantly exposed to large amounts of dust particles and pathogens, and alveolar macrophages are major effector cells in innate host defense against those inhaled irritants by virtue of their phagocytic ability. In addition, alveolar macrophages are central regulators of the resolution of inflammation because of their ability to engulf apoptotic neutrophils. The active phagocytosis of dying cells by macrophages could prevent necrotic cell-causing inflammation and lead to the induction of anti-inflammatory signaling in macrophages by inhibiting the expression of inflammatory cytokines, including CXCL-8 [59]. In contrast, when apoptotic cells are not efficiently cleared by alveolar macrophages, the apoptotic cells go to secondary necrosis and release potentially injurious cytoplasmic contents into the alveolus causing further tissue injury [60, 61]. It was previously reported that MDMs with the Z-AAT allele have impaired efferocytosis [62]. The result of the present study shows that the migratory and phagocytic abilities of MDMs are decreased as the concentration of α-defensins is increased. The concentration of α-defensins is hundreds of times higher in AATD individuals than healthy individuals, and the concentration is increased proportionally with the severity of lung disease in AATD individuals. Findings from previous studies and this study suggest that the phagocytic ability of macrophages to clear dead cells is impaired by the accumulation of Z-AAT and the phagocytosis of invading pathogens is inhibited by excessive amounts of α-defensins in AATD individuals, which could be responsible for chronic inflammation in the lung of AATD individuals.

AAT augmentation therapy with periodic intravenous infusion of pooled human serum AAT is used in individuals with AATD-associated lung diseases. It is very intriguing to examine whether exogenous M-AAT could be used to treat α-defensin-associated lung diseases. To test the possibility, we incubated α-defensin-treated MDMs with M-AAT and found that exogenous M-AAT can improve the migratory and phagocytic abilities of MDMs when the cells are impaired by a high concentration of α-defensins. Because the assay was conducted to determine the therapeutic use of M-AAT on α-defensin-causing macrophage impairment, Z-AAT was not considered to be used for the purpose in the assay. This result implies that AAT augmentation therapy might alleviate α-defensin-associated lung diseases. However, the present study showed that AAT treatment was not sufficient to fully prevent macrophage migration and phagocytosis impairment from α-defensins. Compared with MDM controls, the phagocytic capability of MDMs treated with 2.5 μM of α-defensins was reduced to 50%, and exogenous AAT treatment recovered the MDM phagocytic capability to 75%. Therefore, developing a new therapy which could complement the currently existing AAT augmentation therapy is essential to reduce the burden of α-defensin-associated lung disease in AATD individuals. For this reason, more studies on α-defensins are needed to determine more comprehensive molecular mechanisms underlying α-defensins-impairing macrophage functions and to find therapeutic targets for α-defensin-associated lung diseases.

## Conclusion

Alveolar macrophages play an essential role in innate immunity through efficient clearance of inhaled microbes, damaged tissue, and cells following injury and infection. The present study demonstrates that excessive α-defensins could impair monocyte-macrophage differentiation and therefore their immune function. Due to the general thought that α-defensins are beneficial to host cells, the role of α-defensins in the pathogenesis of AATD-associated lung diseases has been seldom studied. The present study demonstrates that a high concentration of α-defensins suppresses the expression of M2 macrophage markers, inhibits macrophage migration, and impairs macrophage phagocytosis. Also, this study suggests that a high concentration of α-defensins suppresses the expression of M2 macrophage markers by inhibiting ERK1/2 signaling during M- and GM-CSF-derived macrophage differentiation. The findings of this study bring new insights into the pathogenesis of α-defensins in AATD-associated lung diseases and provide the experimental basis for further research on new treatments complementing AAT augmentation therapy, which has been the only specific therapy for AATD individuals for the last three decades.

### Supplementary Information

**Additional file 1: Figure S1. **α-defensins reduce the expression level of CD206 in MDMs. MDMs were differentiated using M-CSF and GM-CSF for seven days and then incubated with different concentrations of α-defensins for 16 h. The expression level of CD206 was compared between MDM controls and α-defensin-treated MDMs for 16 h. Their relative expression is represented by fold change. Statistical analysis was conducted using Wilcoxon test. Statistical significance is denoted by (*) (p-value < 0.05). **Figure S2. α**-defensins have no effect on the phosphorylation of p38. Total proteins were isolated from monocytes, MDM controls, and α-defensin-treated MDMs. **A** Equal amounts of total proteins of the samples were analyzed via SDS-PAGE of phosphor-p38, and **B** protein band intensities of the phosphorylated p38 were compared among the samples. The protein band intensities were measured using NIH ImageJ software, and statistical analysis was conducted using Wilcoxon test. (ns) indicates not significant statistically. **Figure S3**. Phosphorylation of STAT3 positively regulated by ERK1/2. MDMs were incubated with the two different concentrations of U0126, an ERK1/2 inhibitor, overnight. **A** The total proteins were isolated from MDM controls and U0126-treated MDMs and subjected to Western blot assay; the protein levels of total ERK1/2, phosphor-ERK1/2, phosphor-STAT3, and CD163 were compared between MDMs controls and U0126-treated MDMs. **B** Using qRT-PCR, the expression level of CD206 was compared between MDM controls and U0126-treated MDMs. The relative expression of CD206 is represented by fold change. Statistical analysis was conducted using One-way ANOVA. Statistical significance is denoted by (*) (p-value < 0.05).

## Data Availability

The samples, datasets and analysis of this study are available from the corresponding author on reasonable request.
