# Supplementary material for: Alpha-defensins inhibit ERK/STAT3 signaling during monocyte-macrophage differentiation and impede macrophage function
Source: Respir Res. 2023 Dec 11;24:309. doi: 10.1186/s12931-023-02605-0 (PMC10714504; doi:10.1186/s12931-023-02605-0)
Supplement: Supplementary file 1 — Additional file 1: Figure S1. α-defensins reduce the expression level of CD206 in MDMs. MDMs were differentiated using M-CSF and GM-CSF for seven days and then incubated with different concentrations of α-defensins for 16 h. The expression level of CD206 was compared between MDM controls and α-defensin-treated MDMs for 16 h. Their relative expression is represented by fold change. Statistical analysis was conducted using Wilcoxon test. Statistical significance is denoted by (*) (p-value < 0.05). Figure S2. α-defensins have no effect on the phosphorylation of p38. Total proteins were isolated from monocytes, MDM controls, and α-defensin-treated MDMs. A Equal amounts of total proteins of the samples were analyzed via SDS-PAGE of phosphor-p38, and B protein band intensities of the phosphorylated p38 were compared among the samples. The protein band intensities were measured using NIH ImageJ software, and statistical analysis was conducted using Wilcoxon test. (ns) indicates not significant statistically. Figure S3. Phosphorylation of STAT3 positively regulated by ERK1/2. MDMs were incubated with the two different concentrations of U0126, an ERK1/2 inhibitor, overnight. A The total proteins were isolated from MDM controls and U0126-treated MDMs and subjected to Western blot assay; the protein levels of total ERK1/2, phosphor-ERK1/2, phosphor-STAT3, and CD163 were compared between MDMs controls and U0126-treated MDMs. B Using qRT-PCR, the expression level of CD206 was compared between MDM controls and U0126-treated MDMs. The relative expression of CD206 is represented by fold change. Statistical analysis was conducted using One-way ANOVA. Statistical significance is denoted by (*) (p-value < 0.05). [file 12931_2023_2605_MOESM1_ESM.pdf]

**Additional file 1: Figure S1, S2 and S3**

# Alpha-defensins inhibit ERK/STAT3 signaling during monocyte-macrophage differentiation and impede macrophage function.

**Jungnam Lee[1], Naweed Mohammad[1], Yuanqing Lu[1], Regina Oshins[1], Alek Aranyos[1], and Mark Brantly[1*]**

**Figure S1. α-defensins reduce the expression level of CD206 in MDMs.**

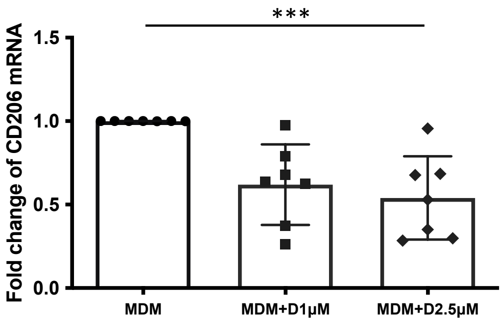


MDMs were differentiated using M-CSF and GM-CSF for seven days and then incubated with different concentrations of α-defensins for 16 hours. The expression level of CD206 was compared between MDM controls and α-defensin-treated MDMs for 16 hours. Their relative expression is represented by fold change. Statistical analysis was conducted using One-way ANOVA. Statistical significance is denoted by (***) (p-value < 0.001).

**Figure S2. α-defensins have no effect on the phosphorylation of p38.**

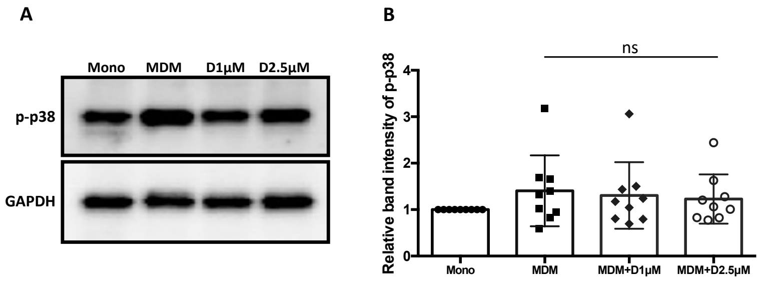


Total proteins were isolated from monocytes, MDM controls, and α-defensin-treated MDMs. (A) Equal amounts of total proteins of the samples were analyzed via SDS-PAGE of phosphor-p38, and (B) protein band intensities of the phosphorylated p38 were compared among the samples. The protein band intensities were measured using NIH ImageJ software, and statistical analysis was conducted using Wilcoxon test. (ns) indicates no statistical difference among the samples.

**Figure S3. Phosphorylation of STAT3 positively regulated by ERK1/2.**

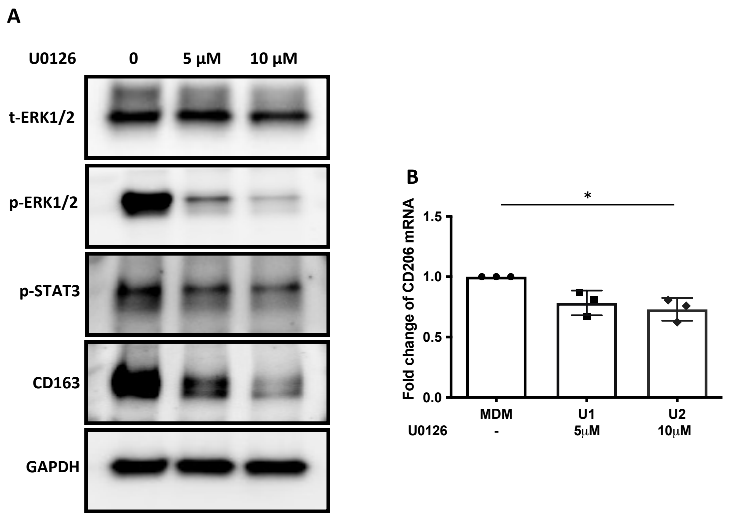


MDMs were incubated with the two different concentrations of U0126, an ERK1/2 inhibitor, overnight. (A) The total proteins were isolated from MDM controls and U0126-treated MDMs and subjected to Western blot assay; the protein levels of total ERK1/2, phosphor-ERK1/2, phosphor-STAT3, and CD163 were compared between MDM controls and U0126-treated MDMs. (B) Using qRT-PCR, the expression level of CD206 was compared between MDM controls and U0126-treated MDMs. The relative expression of CD206 is represented by fold change. Statistical analysis was conducted using One-way ANOVA. Statistical significance is denoted by (*) ($p$-value < 0.05).
